# Forest degradation limits the complementarity and quality of animal seed dispersal

**DOI:** 10.1098/rspb.2022.0391

**Published:** 2022-05-25

**Authors:** Finn Rehling, Jan Schlautmann, Bogdan Jaroszewicz, Dana G. Schabo, Nina Farwig

**Affiliations:** ^1^ Department of Biology, Conservation Ecology, University of Marburg, Karl-von-Frisch-Str. 8, D-35043 Marburg, Germany; ^2^ Faculty of Biology, University of Warsaw, Białowieża Geobotanical Station, PL-17-230 Białowieża, Poland

**Keywords:** environmental niche modelling, forest degradation, old-growth forest, plant–frugivore interactions, functional redundancy, seed dispersal effectiveness

## Abstract

Forest degradation changes the structural heterogeneity of forests and species communities, with potential consequences for ecosystem functions including seed dispersal by frugivorous animals. While the quantity of seed dispersal may be robust towards forest degradation, changes in the effectiveness of seed dispersal through qualitative changes are poorly understood. Here, we carried out extensive field sampling on the structure of forest microhabitats, seed deposition sites and plant recruitment along three characteristics of forest microhabitats (canopy cover, ground vegetation and deadwood) in Europe's last lowland primeval forest (Białowieża, Poland). We then applied niche modelling to study forest degradation effects on multi-dimensional seed deposition by frugivores and recruitment of fleshy-fruited plants. Forest degradation was shown to (i) reduce the niche volume of forest microhabitat characteristics by half, (ii) homogenize the spatial seed deposition within and among frugivore species, and (iii) limit the regeneration of plants via changes in seed deposition and recruitment. Our study shows that the loss of frugivores in degraded forests is accompanied by a reduction in the complementarity and quality of seed dispersal by remaining frugivores. By contrast, structure-rich habitats, such as old-growth forests, safeguard the diversity of species interactions, forming the basis for high-quality ecosystem functions.

## 1. Introduction

Humans have degraded greater than 75% of forest ecosystems worldwide, with much of the remaining old-growth forests at risk of degradation [1–3]. Forest degradation alters the forest's structure, reduces its overall biodiversity and changes species interactions, which may ultimately lead to a functional ‘meltdown’ of ecosystems and the services they provide to humans [4,5]. A key biotic function of ecosystems is seed dispersal by frugivorous animals, as it determines the spatial distribution and genetic composition of plants at small and large scales [6,7]. Forest degradation results in a loss of frugivores and changes plant–frugivore interactions [7,8]. Whether the frugivore loss will also affect seed dispersal of plants, depends on how specialized interactions between plants and frugivores are [9]. When plant–frugivore interactions are highly specialized, interactions can hardly be compensated by other species. The frugivore loss will then often lead to the loss of interactions, with detrimental effects on seed dispersal of the respective plants [8,9]. By contrast, when seed dispersal is characterized by a low specialization between plants and frugivores [10–12], seed dispersal may be relatively robust to forest degradation as interactions of sensitive species can be compensated by other less sensitive, generalist species [12–14].

Most research on forest degradation effects on seed dispersal has been limited to quantitative investigations of seed dispersal (i.e. assessments of number of removed fruits). The effectiveness of seed dispersal by frugivores, however, is also influenced by qualitative factors, which determine the probability of a seed to produce a mature plant in a given habitat [15]. Reductions in seed dispersal effectiveness through changes in qualitative components may remain undetected, when generalist frugivores quantitatively compensate the loss of fruit removal by specialists in degraded habitats.

Forest degradation may directly affect the quality of seed dispersal, when certain microhabitats change their characteristics, or completely disappear, and these changes in microhabitat structure affect regeneration of plants [16,17]. In addition, forest degradation may affect seed dispersal quality indirectly through changes in the behaviour of frugivores that will ultimately affect spatial pattern of seed deposition [18]. Not only frugivores, but also seed-predating animals or herbivores similarly adjust their behaviour in degraded forests, influencing the regeneration of plants [16,19,20]. Thus, effects of forest degradation on the structure of microhabitats, and the behaviour of frugivores and other animals affect two important components of seed dispersal quality, i.e. the spatial pattern of seed deposition and the recruitment of plants after deposition.

The manifold pathways of how forest degradation can affect the quality of seed dispersal by frugivores make predictions on the direction and magnitude of effect sizes difficult [21]. Reductions in the quality of seed dispersal may occur at species-level, when interactions of high-quality seed dispersers are quantitatively compensated by non-equivalent (i.e. complementary), low-quality seed dispersers [22–24]. In addition, reductions in seed dispersal quality can occur within species, when individuals of a certain frugivore are present in both, old-growth and degraded forests, but the seed dispersal quality is reduced in the latter one; referred to as ‘cryptic functional loss' of species [25]. Changes of frugivore behaviour in degraded habitats should often result in reductions of the quality of seed deposition and plant recruitment [25]. However, qualitative investigations of seed dispersal in old-growth and degraded forests are rare due to the challenges in tracking frugivore effects on late stages of seed dispersal in complex environments [26,27]. Understanding the mechanisms of how forest degradation affects the quality of seed dispersal will help to identify key forest microhabitat characteristics and species that disproportionally contribute to ecosystem functioning.

In this study, we investigated the effect of forest degradation on seed dispersal of a diverse plant–frugivore community in the temperate lowland forest of Białowieża, Poland. We disentangled the complex pathways of how forest degradation influences the quality of plant–frugivore interactions, by studying (i) the structure of forest microhabitats, (ii) the structure of deposition sites of the three frugivore species most important for fruit removal in Białowieża Forest [28] (i.e. *Sylvia atricapilla*, *Turdus merula* and *T. philomelos*) and all remaining dispersers combined and (iii) the effect of forest microhabitat characteristics on recruitment of the fleshy-fruited plant community in old-growth and degraded forests. However, the suitability of microhabitats for seed deposition and plant regeneration is the net outcome of multiple factors that can differ in their effects on plant life stages [21]. To account for the multi-dimensional character of seed dispersal quality, we focused on three characteristics of forest microhabitats important for seed dispersal and plant recruitment to the adult stage. We recorded the canopy cover as an inverse proxy for light availability [29], the ground vegetation as a combined, inverse proxy for seed predation and early plant competition [30,31], and the volume of deadwood as an inverse proxy for the intensity of herbivory [20]. We then modelled multi-dimensional niches [32] to characterize the structure of forest microhabitats and deposition sites of frugivores.

We sought answers to the following questions. (i) How does forest degradation affect the structure of forest microhabitats (canopy cover, ground vegetation, deadwood)? (ii) What is the impact of forest degradation on seed deposition by the frugivore community and by frugivore species therein? (iii) Do frugivores disperse seeds non-randomly depending on the forest type (i.e. in contrast with what would be expected based on the availability of microhabitats)? (iv) Do frugivores deposit seeds in microhabitats with the same characteristics in old-growth and degraded forest, either among species (i.e. redundant versus complementary depositions) or within species (i.e. cryptic functional loss)? (v) How does forest degradation, canopy cover and ground vegetation affect the early stages of regeneration of plants?

## 2. Material and methods

### (a) Study area and sites

The study was conducted in Białowieża Forest, which spans the borders of Poland and Belarus and is the last old-growth forest in European lowlands. The Polish part of the forest (approx. 675 km²) is divided into Białowieża National Park (approx. 105 km²) and forests managed by the Polish National Forest Holding ‘State Forests’. The forest is structurally rich and consists of a mosaic of unevenly aged, species-rich tree stands as well as a large amount of deadwood [33]. The Białowieża Forest was first protected as a royal hunting area, before a 47.5 km² area within the current Białowieża National Park was declared as a national park. As such, this small area has remained largely pristine for roughly 500 years, with only minor disturbances by humans [33]. By contrast, in the forests managed by the Polish National Forest Holding ‘State Forests’, commercial logging has been conducted since the First World War [3,33]. Our study was conducted at 12 of 17 sites previously used in studies of plant–frugivore interactions in ash–alder floodplain forests [28,34]. The 12 sites were scattered over approximately 400 km², covering two-thirds of the Polish part of Białowieża Forest (electronic supplementary material, table S1). Four study sites were situated in Białowieża National Park (stand age: approximately 70–150 years) and eight in the managed forests (stand age: approximately 60 years), referred to, respectively, herein as ‘old-growth’ and ‘degraded’ forest.

### (b) Study species

Fourteen commonly occurring woody plant species producing fleshy fruits were included in this study: *Cornus sanguinea, Euonymus europaeus, Frangula alnus, Prunus padus, Rhamnus cathartica, Ribes alpinum, R. nigrum, R. spicatum, Rubus fruticosus agg., R. ideaus, Sambucus racemosa, S. nigra, Sorbus aucaparia* and *Viburnum opulus.* The fruiting season of this plant community starts in mid-June, with *P. padus* and *R. spicatum*, and ends in mid-October, with *E. europaeus*. At least 41 animal species act as seed dispersers within the study sites [11,28,34]. In this study, we only examined the seed deposition pattern by *S. atricapilla*, *T. merula* and *T. philomelos* as the sample size for statistical analysis was large enough only for these frugivores, and all other dispersers combined (see ‘Creating niche spaces'). These three bird species are small-bodied, generalist frugivores, and are among the quantitatively most important frugivores in Białowieża Forest [28,34] and throughout Europe [35].

### (c) Seed deposition

The spatial seed deposition by frugivores in Białowieża Forest were assessed along five transects per study site from 2016 to 2018. These transects had a length of 100 m and were separated from each other by at least 20 m. Scats with seeds of the plant species found within a range of 1 m to the left and right of each transect (total area of 1000 m² per site) were collected. During the fruiting period, the transects were checked every 10 days for new scats. This resulted in 11 repetitions of the transect walks in 2016 and 2018, and, due to a shorter fruiting season, 9 repetitions in 2017. In the case of heavy rainfall events, scats were collected at least 2 days after they had ended. We collected approximately 4000 seed-containing scats. Seeds recovered from scats were not mechanically damaged [34]. Scats of most mammals were identified in the field; otherwise, they were stored in sterile tubes at −20°C and analysed following extraction of their DNA. A modification of the barcoding protocol [36] was used to extract DNA of animal origin from the surfaces of deposited seeds (success rate = 90.1%). Additional information on disperser identification using DNA barcoding is provided in electronic supplementary material, text S1.

### (d) Seedling recruitment

We performed sowing experiments with nine fleshy-fruited plant species at four study sites (two in degraded forest and two in old-growth forest) that we also used for the study of seed deposition. Each year, we collected fruits of at least six conspecific adults of the nine most abundant plant species (*E. europaeus, F. alnus, P. padus, R. cathartica, R. nigrum, R. spicatum, S. nigra, S. aucaparia* and *V. opulus*) in each forest type. We removed the pulp, dried the seeds for 48 h at room temperature and mixed them afterwards. At each study site, we established 10 plots with independent species-specific 50 × 50 cm² subplots for 3 years of sowing experiments; 2016 (*n* = 40 subplots), 2017 (*n* = 20) and 2018 (*n* = 40). We sowed 25 seeds per subplot. *Sambucus nigra* did not occur in the old-growth forest and was only studied in the degraded forest. Due to late frosts in spring, *P. padus* did not produce any fruits in 2016 and 2017 and was only studied in 2018. This resulted in 2500 sown seeds for seven of the nine plant species, 1250 for *S. nigra* (only in degraded forest) and 1000 for *P. padus* (only in 2018). We found negligible external seed input in sowing experiments, when we checked for emerging seedlings on a control subplot next to each subplot. An extended description of the seedling recruitment experiments is provided in electronic supplementary material, text S2.

To investigate first-year survival of seedlings, we tracked the survival of emerging seedlings from sowing experiments. In addition, we searched for seedlings at eight study sites, that we used for studies on seed deposition of the frugivore–plant community from 2017 to 2019 [34]. We only considered seedlings with cotyledons. We were able track the survival of 477 seedlings as part of the sowing experiment, and 101 seedlings that ‘naturally’ occurred in the forest.

### (e) Assessing microhabitat characteristics

To identify microhabitat characteristics of deposition sites of frugivores, each transect was split into five segments, each of which was 20 m long, and each deposited scat was then assigned to the closest segment. At each of these segments, the following microhabitats characteristics were recorded: canopy cover, ground vegetation cover and the volume of deadwood. Canopy cover was determined based on up to six (5.4 ± 0.49) hemispherical photos taken at the centre of each segment using a fish-eye lens. All photos were taken during the fruiting period from June to October in 2016 and 2017, and the area covered by forest canopy was then analysed using DHPT 1.0 software [37]. Canopy cover was calculated as the mean of the forest coverage as depicted in taken photos. In addition, the relative coverage of vegetation at heights of 0 m, 0.5 m and 1 m located within a radius of 10 m around the centre of each transect segment was estimated. Ground vegetation was calculated as the mean of the mean vegetation cover across the different heights in 2016 and 2017. The amount of deadwood was determined within a range of 5 m to the right and left of the transect, by measuring the diameter and length of all dead tree logs with a minimum diameter of 10 cm (at half of the length of tree log) and the diameter and height of all tree stumps with a minimum height of 50 cm. The measurements were made in August 2017. The volume of deadwood was calculated as the sum of standing and lying deadwood per transect segment. At the 40 plots used for the sowing experiment, and at locations where we investigated the survival of first-year seedlings, we used the same method to estimate canopy cover and ground vegetation as for the deposition sites of frugivores. However, because the field period was very labour-intensive, we performed the microhabitat assessment at these locations only up to three times and used the mean values for the analyses.

### (f) Creating niche spaces

The analyses were conducted using a hypervolume approach with a Gaussian kernel density estimation, which allowed a relatively loose fit of data by the hypervolume and the inclusion of data points away from the centroid [32,38]. Standardized canopy cover, ground vegetation and the volume of deadwood were used as environmental dimensions of the hypervolume to assess the niche space of microhabitats in old-growth and degraded forest (forest microhabitat space) and deposition sites by frugivores therein (deposition microhabitat space). We separately examined only the seed deposition by *S. atricapilla*, *T. merula* and *T. philomelos*, and for the 20 other frugivores combined, as the number of replicates was high (*n* > 60) that was needed to reduce uncertainty in the three-dimensional niche spaces. In addition, the deposition microhabitat space of the frugivore community was assessed in old-growth and degraded forest, by combining the microhabitat spaces of the three frugivore species and the mixed dispersers in old-growth and degraded forest, respectively. The resulting niche spaces were used to describe two independent properties influencing the quality of microhabitats for plant growth and therefore the quality of seed dispersal by frugivores [15]: (i) the volume of the microhabitat space (i.e. the niche space of available microhabitats), which was expected to correlate with the number of microhabitats supporting the persistence of plant species over time and (ii) the centroid of the niche space, which was expected to directly influence the probability of a seed to produce a mature plant. Additional information on the creation of niche spaces is provided in electronic supplementary material, S3. Uncertainties in the microhabitat spaces of the forest type and in the deposition of frugivores were determined by bootstrapping (*n* = 200) the subsamples without replacement. For each bootstrap, we then created the forest microhabitat spaces and the deposition microhabitat spaces of frugivore species and the frugivore community as described above. A constant bandwidth over all bootstraps was maintained using the mean of the Silverman estimates of all combinations of forest type × frugivore × bootstrap subsample for the forest microhabitat space and the deposition microhabitat space of frugivore species, respectively.

### (g) Data analyses

#### (i) Niche spaces

Statistical analyses of niche spaces remain a challenge [38], and to our knowledge, the use of an integrated approach is currently not possible. Thus, instead, the volumes and centroids of the bootstrapped subsamples of the forest and seed deposition microhabitats were compared pair-wise to determine the probability that the value of one group was larger or smaller than that of another group (based on two-tailed *p*-values). Additional details on comparisons of the volumes and centroids of the forest and deposition microhabitat spaces are provided in the electronic supplementary material, text S3. As small differences in the centroids of forest and deposition microhabitat spaces strongly affected the recruitment of seedlings of certain plant species (see results), we did not include a Bonferroni correction for multiple pair-wise testing to avoid a type-II error [39]. To study the complementarity of seed deposition by frugivore species in old-growth and degraded forest, we calculated the fraction of the total volume of the deposition microhabitat space of frugivore community that was filled either by frugivore species or by different combinations of frugivore species.

#### (ii) Seedling recruitment

To analyse the effect of the interaction between microhabitat dimensions and forest type on plant recruitment, we used a mark-recapture design which assumes that all seeds in the soil remain viable throughout the study period, i.e. if seeds had not recruited in the first year, seeds were able to recruit in the next year. However, we found that seed bank dynamics were very variable among plant species. Especially, *S. nigra* recruited very poorly overall (*n* = 4 seedlings, approximately 0.3% germination rate). To allow a full-factorial approach of analysing the data, we entirely excluded *S. nigra* from the analyses, pooled the data from the sowing experiment among years (2016, 2017 and 2018) and analysed only the year of peak recruitment for each plant species (the first year since sowing in seven species, the second year since sowing in *E. europaeus* and *V. opulus*). We used a generalized linear mixed model (binomial error distribution and a logit link) with the number of seedlings in the year of peak recruitment (successes) and the number of seeds that had remained in soil until then (failures) as a response variable (binomial denominator). We used the standardized canopy cover, the standardized ground vegetation, the forest type (old-growth versus degraded) and their interactions as fixed factors in this model. We included a maximal random effects structure by including random intercepts for species and plots within study sites, and random slopes for species responses to forest type. In these models, we also included observation level random effects to account for overdispersion.

To analyse the effect of the interaction between microhabitat dimensions and forest type on early survival of seedlings, we used a generalized linear mixed model (binomial error distribution and a logit link) with survival as a binary response variable, the standardized canopy cover, standardized ground vegetation, forest type (old-growth versus degraded) and their interactions as fixed factors, and study site as a random effect. To allow a non-zero fit of the data, we pooled seedlings from different study years. We included only five plant species with a sufficient number of replicates: *E. europaeus* with 179 seedlings, *F. alnus* with 54 seedlings, *R. spicatum* with 155 seedlings, *S. aucuparia* with 127 seedlings and *V. opulus* with 63 seedlings. The number of species was too low to estimate random intercepts and slopes. Our analysis thus included interactions of plant species with canopy cover, ground vegetation and forest type as fixed factors in the analysis. Additional information on the data analysis of plant recruitment is provided in the electronic supplementary material, text S2.

All statistical analyses were performed using R v. 4.1.1 [40], and the hypervolumes were created using the package ‘hypervolume’ v. 2.0.12 [41]. Generalized linear mixed models were constructed using the package ‘glmmTMB’ v. 1.0.2.1 [42]. Significance values for the effect of fixed factors were obtained using Wald-*χ*^2^ tests in the package ‘car’ v. 3.0-9 [43]. The performance of all models was evaluated using the package ‘DHARMa’ v. 0.4.3 [44].

## 3. Results

### (a) Effect of forest degradation on microhabitat structure of the forest, and deposition sites of frugivore community

**Figure 1.**
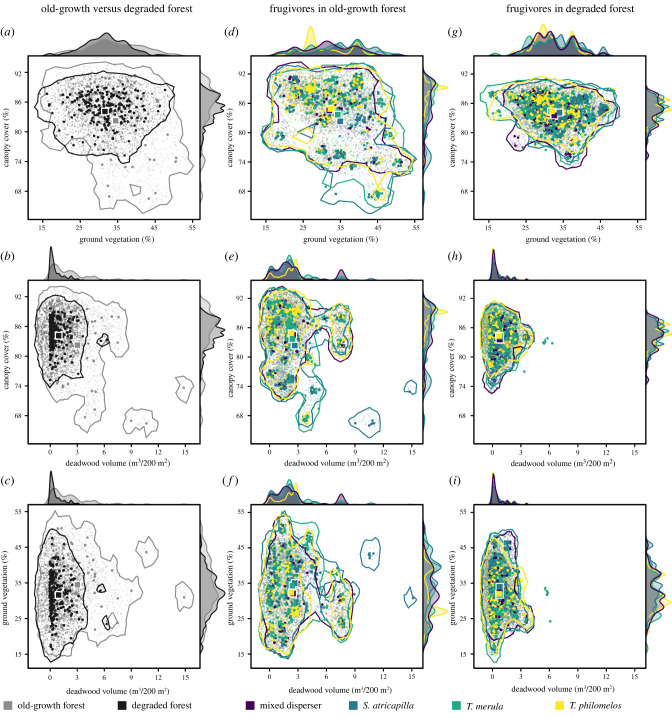
The three-dimensional forest microhabitat space of old-growth and degraded forest in Białowieża Forest (*a*–*c*), and the deposition microhabitat space of frugivores (mixed dispersers, *S. atricapilla*, *T. merula* and *T. philomelos*) in old-growth (*d*–*f*) and degraded (*g*–*i*) forest, illustrated by two-dimensional representations of (*a*,*d*,*g*) canopy cover and ground vegetation, (*b*,*e*,*h*) canopy cover and the volume of deadwood and (*c*,*f*,*i*) ground vegetation and the volume of deadwood. Circles represent the transect segments of the forest (*a*–*c*) or the deposited seeds of the frugivores therein (*d*–*i*). The centroids (squares) indicate the centre of the forest (*a*–*c*) and the deposition microhabitat spaces (*d*–*i*) along each of the three microhabitat characteristics (canopy cover, ground vegetation and volume of deadwood). (Online version in colour.)

**Figure 2.**
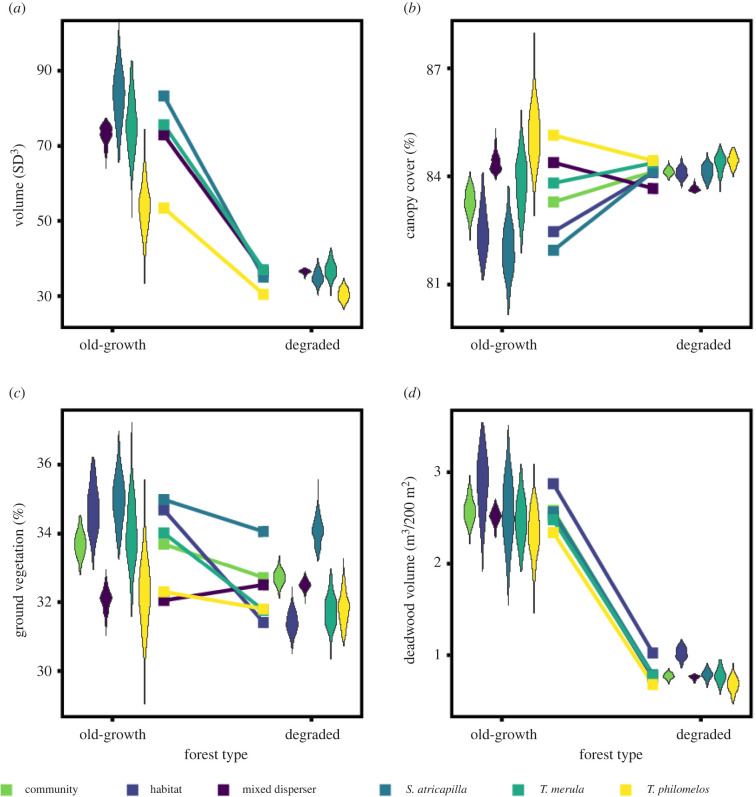
(*a*) Volume of the deposition microhabitat space of frugivore species (*S. atricapilla, T. merula* and *T. philomelos*, mixed dispersers) in Białowieża Forest, Poland. (*b–d*) Centroids of the forest microhabitat spaces ('habitat') and the deposition microhabitat spaces of the combined frugivore community ('community') and of frugivore species (*S. atricapilla, T. merula* and *T. philomelos*, mixed dispersers) along the three environmental dimensions of forest microhabitats in old-growth and degraded forest: (*b*) canopy cover, (*c*), ground vegetation and (*d*) volume of deadwood. (Online version in colour.)

The degraded forests were characterized by correlated changes, both in the niche volume and the characteristics of the forest microhabitats and of deposition sites by the frugivore community therein. The volume of the three-dimensional forest microhabitat space was 2.4 times larger in old-growth than in degraded forest (118 SD³ versus 50 SD³; figure 1). This result could be attributed to the rarity in degraded forest of forest gaps with a very low canopy cover and of microhabitats with a large amount of deadwood (figure 1). In general, old-growth forest had a larger amount of deadwood (centroid: 143.5 versus 51.0 m³ ha^–1^, *p* < 0.01), denser ground vegetation (centroid: 34.7 versus 31.4%, *p* < 0.01) and lower canopy cover (centroid: 82.5 versus 84.1%, *p* = 0.03) than degraded forest (electronic supplementary material, table S3). The volume of the deposition microhabitat space of the frugivore community was approximately 2.7 times larger in old-growth than in degraded forest (102 SD³ versus 38 SD³). The centroids of the forest microhabitat space in both forest types were largely equal to those of the deposition microhabitat space of the frugivore community (four of six pair-wise comparisons, *p* > 0.05; figure 2; electronic supplementary material, table S4). Only in degraded forest, the frugivore community preferentially deposited seeds within microhabitats with a lower amount of deadwood and a denser ground vegetation (two of six pair-wise comparisons, *p* < 0.01; figure 2; electronic supplementary material, table S4). This was in contrast with what was expected based on the availability of forest microhabitats (i.e. non-random seed dispersal at the community level).

### (b) Effect of forest degradation on microhabitat structure of deposition sites of different frugivore species

**Figure 3.**
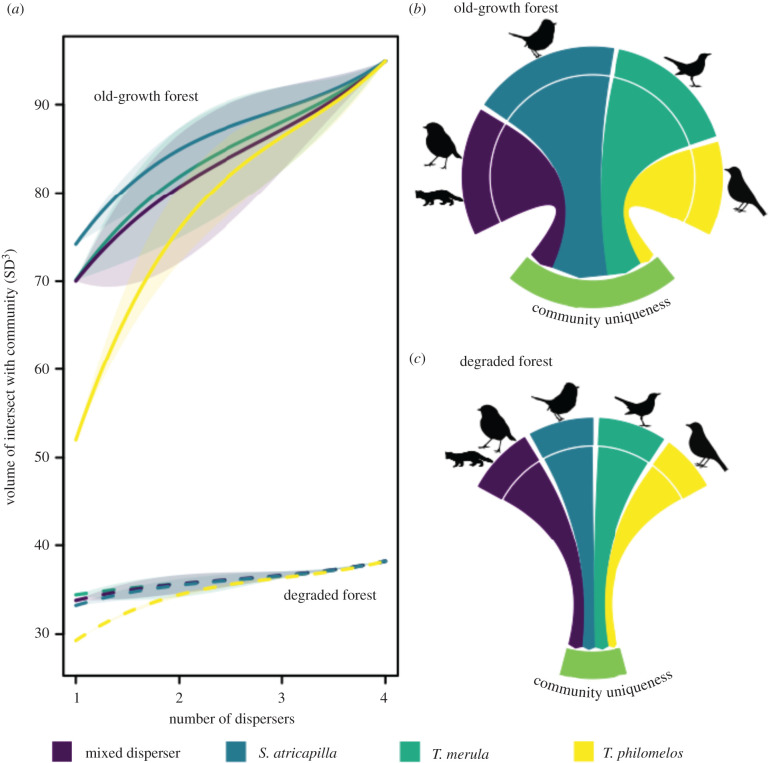
(*a*) Volume of the deposition microhabitat space in old-growth (solid lines) and degraded (dotted lines) forest as a function of different combinations of frugivore species (*S. atricapilla, T. merula* and *T. philomelos*, mixed dispersers) in Białowieża Forest, Poland. The lines indicate the mean volume of the bootstrapped deposition microhabitat spaces for each combination of frugivore species, and the shaded areas the best and worst combinations of the different frugivore species. (*b,c*) The volume and uniqueness of the deposition microhabitat space of the different frugivores, and the uniqueness of the community in the (*b*) old-growth and (*c*) degraded forest. The upper part of the chord-diagram shows the niche space of seed deposition (i.e. the volume of the microhabitat space) of the different frugivores; the lower chord-diagram shows the proportion of each frugivore's deposition to community uniqueness (i.e. part of the microhabitat space only filled by one frugivore) and the summed proportions of the uniqueness of depositions of frugivore species (‘community uniqueness', light green).

At the level of frugivore species, the volume and centroid of the deposition microhabitat space followed complex patterns in the two forest types (figures 1–3). The deposition microhabitat spaces of *S. atricapilla*, *T. merula*, and the mixed dispersers were generally larger than those of *T. philomelos*, independent of forest type (*p* ≤ 0.04; figure 3; electronic supplementary material, table S5). Furthermore, in old-growth forest, *S. atricapilla* deposited seeds more frequently in microhabitats characterized by a high light availability and a dense ground vegetation than the mixed dispersers and *T. philomelos* (figure 2; *p* ≤ 0.01; electronic supplementary material, tables S6–S8). Independent of these complex patterns at species level, among-frugivore differences in the centroids of deposition microhabitat spaces were always larger in old-growth than degraded forest, i.e. for the canopy cover (3.2% versus 0.78%), ground vegetation (2.94% versus 2.3%) and amount of deadwood (0.23 versus 0.11 m³/200 m²). Similarly, the seed deposition varied more strongly within frugivores in old-growth than degraded forest along each microhabitat characteristic (12 of 12 comparisons of the SD of frugivore centroids between old-growth and degraded forest; figure 2*b–d*; electronic supplementary material, tables S7 and S8). The variation in seed deposition within frugivores was on average 3.8, 2.1 and 4.2 times larger (mean = 3.4) along the canopy cover, ground vegetation and deadwood gradient, respectively (figure 2).

### (c) (Non-)random seed deposition by frugivore species

Seed deposition by frugivores was often non-random at the level of species (11 of 24 pair-wise comparisons, *p* ≤ 0.04; electronic supplementary material, table S9). The frequent seed depositions of mixed dispersers and *T. philomelos* in microhabitats with a high canopy cover and less dense ground vegetation did not correspond to microhabitat availability, especially in old-growth forest (four of four pair-wise comparisons, *p* ≤ 0.04) but also often in degraded forest (two of four pair-wise comparisons, *p* ≤ 0.03). In degraded forest, *S. atricapilla* deposited seeds significantly more often in microhabitats with a dense ground vegetation (*p* < 0.01). Similarly, in degraded forest, all frugivore species deposited seeds in microhabitats with a smaller volume of deadwood with a higher frequency than would be expected by chance (*p* ≤ 0.01). Otherwise, the seed deposition patterns in both forest types corresponded to the availability of microhabitat with certain characteristics, namely random seed deposition by frugivore species (13 of 24 pair-wise comparisons, *p* > 0.05; electronic supplementary material, table S9).

### (d) Complementarity versus redundancy of seed depositions

In old-growth forest, 50–70% of the volume of the deposition microhabitat space of the community was filled by one frugivore species, with additional frugivores contributing an average of 15.0%, 7.3% and 7.5% (figure 3). The deposition microhabitat space of old-growth forest was consistently reduced when *T. philomelos* was part of the frugivore combination, because the deposition microhabitat space of this species was the smallest and it had the highest overlap with that of other frugivores (two of three pair-wise comparisons, *p* ≤ 0.03, one comparison, *p* = 0.07; figures 2*a* and 3; electronic supplementary material, table S10). In degraded forest, the deposition microhabitat space of one frugivore accounted for 86% of the total volume of the community, and any combination of two frugivores resulted in a mean completeness of 92–96% of the total volume of the deposition microhabitat space.

### (e) Recruitment of fleshy-fruited plants in forest microhabitats

**Figure 4.**
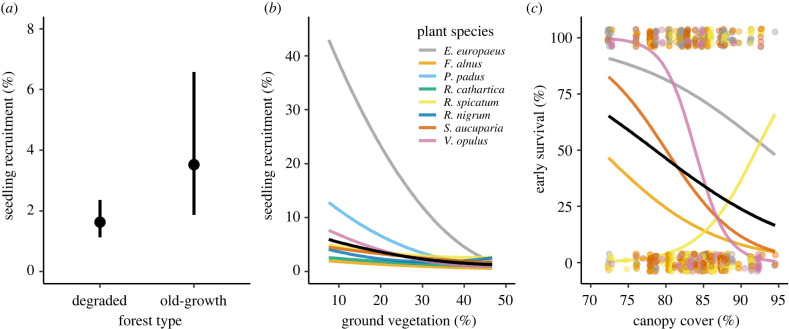
Seedling recruitment in the peak year and early survival of the fleshy-fruited plant community in Białowieża Forest, Poland. (*a*) The effect of forest type (old-growth versus degraded) on seedling recruitment. Mean ± 95%CI. (*b*) The relationship between ground vegetation and seedling recruitment. (*c*) The relationship between canopy cover and early survival of the seedlings. In (*b*,*c*), the black line represents the predicted mean of the plant species, and the coloured lines represent the mean of the plant species. (Online version in colour.)

Seedling recruitment of the plant community was higher in old-growth than degraded forest (*χ*^2^ = 7.21, *p* = 0.007; figure 4*a*) and increased in microhabitats with a less dense ground vegetation (*χ*^2^ = 10.64, *p* = 0.001; figure 4*b*). Seedling recruitment was not related to canopy cover (*χ*^2^ = 2.20, *p* = 0.138), and there was no interaction between canopy cover, ground vegetation cover and forest type (see electronic supplementary material, table S11). For a subset of five plant species, early survival of recruits differed among plant species (*χ*^2^ = 45.34, *p* < 0.001), spanning from *R. spicatum* with a survival probability of 5.5% to *E. europaeus* with a survival probability of 76.6%. For four plant species (*E. europaeus*, *F. alnus*, *S. aucuparia* and *V. opulus*), early survival of seedlings was higher in light environments, but early survival of *R. spicatum* increased with canopy cover (canopy cover × plant species interaction, *χ*^2^ = 18.82, *p* = 0.001; figure 4*c*). Forest type, ground vegetation cover and their interactions with canopy cover and plant species did not affect early survival of the plants (electronic supplementary material, table S11).

## 4. Discussion

This study revealed that forest degradation affected the effectiveness of seed dispersal through changes in two qualitative components of seed dispersal: seed deposition by frugivores and recruitment of plants. Forest degradation simplified the microhabitat structure by reducing the available niche volume and altering characteristics of forest microhabitats. The observed changes in microhabitat structure from old-growth to degraded forests led to a correlated simplification of the microhabitat structure of seed deposition sites of frugivores in degraded forest. In particular, forest microhabitats with a high light availability and a large amount of deadwood were lost due to forest degradation. In addition, among- and within-frugivore differences in seed deposition were reduced in degraded forest. Altogether, consequently, forest degradation reduced the complementarity of seed deposition by frugivores. We propose that this loss in complementarity was accompanied by a reduction in the quality of seed dispersal as (i) a low diversity of seed deposition sites and (ii) the reduced availability of light and deadwood restrains plant regeneration [20,45]. Accordingly, our study showed that forest degradation directly reduced seedling recruitment of the fleshy-fruited plants, and lower light availability reduced early survival of seedlings. Thus, forest degradation may ultimately reduce population growth of plants, especially of light-demanding species, thereby altering plant composition compared to old-growth forest [17,33,45].

Our findings are a further demonstration of seed dispersal as a rather generalized plant–animal interaction [10–12], as seed deposition sites of small-bodied key frugivores are broadly similar. In Białowieża Forest, the fleshy-fruited plant community is mostly associated with one habitat type (i.e. ash–alder forest). In ash–alder forests, the microhabitat use of most animals may not be limited as animals can move freely and are rarely restrained by within-habitat boundaries. Only large mammals tend to avoid microhabitats with large tree logs when predators are present as the tree logs block escape routes [20]. Small differences in microhabitat use by frugivores in forests could explain why the seed deposition sites of frugivores largely overlapped in their characteristics and why no frugivore played an outsized role in seed deposition within microhabitats with a certain characteristic. Similarities in microhabitat use by frugivores also explain why frugivore abundance is usually more important than frugivore diversity for seed deposition in temperate forests [46,47]. By contrast, in study systems where seed dispersal across habitat boundaries is frequent (e.g. forest versus meadow), differences in the spatial seed deposition among frugivores were pronounced, even among the small-bodied key frugivores in this study [22,48].

Nonetheless, the small differences between the deposition patterns of certain frugivore species at the local scale, as demonstrated herein for *S. atricapilla* (centroid = 81.95%) and *T. philomelos* (85.15%) along the canopy cover of old-growth forest, can lead to large increases in early survival of seedlings (15% on average, up to 44% in *V. opulus*). In addition, seed deposition pattern by *T. philomelos* was often non-random, the least diverse and most redundant of all frugivores in the three microhabitat characteristics. Thus, although seed deposition by frugivores in temperate forests is rather generalized, there are subtle differences between frugivores that are important for plant recruitment. If these frugivore effects persist until plants reach maturity [26], small-bodied frugivores will ultimately differ in the quality of seed dispersal for the plant species and community [15]. However, abiotic and biotic factors influencing the suitability of forest microhabitats for plant regeneration can be context-dependent [21]. The magnitude and direction of environmental effects on plant growth may change over time as plants grow and the habitat changes [21]. Simultaneously, the need to pool all rare dispersers into a single group of mixed dispersers may have obscured more subtle differences between the contributing frugivore species, especially that of mammals. Due to these limitations, it is yet not clear how much the effects of forest degradation on seed dispersal quality will limit the effectiveness of seed dispersal over the full life cycle of plants, and whether frequent dispersers or rare, high-quality dispersers will be most important for plant populations and communities in old-growth and degraded forests.

Frugivores were functionally complementary in old-growth forest, but redundant in degraded forest. In isolation, the high redundancy of seed dispersal in degraded forests would seem to indicate its robustness towards forest degradation. Yet it should instead be interpreted as an increased homogenization of seed dispersal compared to old-growth forests. It ultimately results from a decrease in structural heterogeneity of the habitat, species loss and an alignment of plant–frugivore interactions between and within the remaining species. Structural complexity of habitats promotes highly diverse species interactions [49]. This increases the probability that species with strongly divergent optima in terms of habitat conditions or interaction partners can co-occur [50,51]. A decrease in the functional complementarity of seed dispersal may thus pose a challenge to the population dynamics of plant species in degraded forests and modify the community composition and functioning of forest ecosystems.

Our study is one of the very few to show the shift in an ecosystem function that accompanies forest degradation. This was evidenced by a shift from the high-quality, partially complementary outcome of plant–frugivore interactions in complex habitats to the low-quality, almost entirely redundant outcome of plant–frugivore interactions of the same species in degraded habitats. These observations highlight the importance of heterogeneous habitats, such as old-growth forests [5], in maintaining biodiversity and species interactions. Furthermore, it stresses the need to ensure structural heterogeneity, not only at the landscape scale [52] but also at the local scale, in order to preserve the ecosystem functions of forests. The species losses that commonly follow anthropogenic disturbances imply a simultaneous loss of the complementary functions provided by other co-occurring species [25]. The assumption that functional redundancy will protect species and ecosystem functions can thus be erroneous, if the original distributions and interactions of species are unknown [53]. Our study demonstrates the need to integrate qualitative aspects into assessments of the effects of forest degradation on biodiversity, ecosystem functionality and the ecosystem services provided to humans.

## Data Availability

The original contributions and R code presented in the study are included in the article, electronic supplementary material [55] or Dryad Digital Repository [54].
